# The global incidence and trends of three common flavivirus infections (Dengue, yellow fever, and Zika) from 2011 to 2021

**DOI:** 10.3389/fmicb.2024.1458166

**Published:** 2024-08-14

**Authors:** Yuanhao Liang, Xingzhu Dai

**Affiliations:** ^1^Clinical Experimental Center, Jiangmen Engineering Technology Research Center of Clinical Biobank and Translational Research, Jiangmen Central Hospital, Jiangmen, China; ^2^Department of Stomatology, Guangdong Provincial People’s Hospital (Guangdong Academy of Medical Sciences), Southern Medical University, Guangzhou, China

**Keywords:** global burden of disease, flavivirus infections, dengue, yellow fever, Zika infection, age-standardized incidence rate, estimated annual percentage change importance

## Abstract

**Background:**

Flavivirus pose a continued threat to global health, yet their worldwide burden and trends remain poorly quantified. We aimed to evaluate the global, regional, and national incidence of three common flavivirus infections (Dengue, yellow fever, and Zika) from 2011 to 2021.

**Methods:**

Data on the number and rate of incidence for the three common flavivirus infection in 204 countries and territories were retrieved from the Global Burden of Diseases, Injuries, and Risk Factors Study (GBD) 2021. The estimated annual percent change (EAPC) was calculated to quantify the temporal trend during 2011–2016, 2016–2019, and 2019–2021, respectively.

**Results:**

In 2021, an estimated 59,220,428 individuals were infected globally, comprising 58,964,185 cases of dengue, 86,509 cases of yellow fever, and 169,734 cases of Zika virus infection. The age-standardized incidence rate (ASIR) of the three common flavivirus infections increased by an annual average of 5.08% (95% CI 4.12 to 6.05) globally from 2011 to 2016, whereas decreased by an annual average of −8.37% (95% CI −12.46 to −4.08) per year between 2016 to 2019. The ASIR remained stable during 2019–2021, with an average change of 0.69% (95% CI −0.96 to 2.37) per year globally for the three common flavivirus infections. Regionally, the burden of the three common flavivirus infections was primarily concentrated in those regions with middle income, such as South Asia, Southeast Asia, and Tropical Latin America. Additionally, at the country level, there was an inverted “U” relationship between the SDI level and the ASI. Notably, an increase in the average age of infected cases has been observed worldwide, particularly in higher-income regions.

**Conclusion:**

Flavivirus infections are an expanding public health concern worldwide, with considerable regional and demographic variation in the incidence. Policymakers and healthcare providers must stay vigilant regarding the impact of COVID-19 and other environmental factors on the risk of flavivirus infection and be prepared for potential future outbreaks.

## 1 Introduction

Flaviviruses are single-stranded, positive-sense RNA viruses that are transmitted by insect vectors, and they belong to one of the four genera within the family Flaviviridae (Gould and Solomon, 2008). Over recent decades, notable flaviviruses such as Dengue virus (DENV), West Nile virus (WNV), Zika virus (ZIKV), and yellow fever virus (YFV) have been responsible for the emergence and re-emergence of numerous infectious diseases, posing enduring threats to global health (Pierson and Diamond, 2020). Flavivirus infections can be broadly categorized into two phenotypes: visceral and neurotropic. DENV and YFV typically cause systemic diseases involving hemorrhage, while WNV and ZIKV can result in severe neurological complications (Gould and Solomon, 2008; Pierson and Diamond, 2020). Additionally, ZIKV possesses a unique ability to infect the reproductive tract, facilitating sexual transmission and allowing the virus to reach the developing fetus. This can result in microcephaly, congenital malformations, and even fetal demise (Pierson and Diamond, 2020).

DENV is recognized as the fastest-spreading mosquito-borne virus, threatening roughly half of the global population with infection (Brady et al., 2012; Messina et al., 2019). Based on data from the lobal Burden of Diseases, Injuries, and Risk Factors Study (GBD) 2019, there were an estimated 56.9 million cases of dengue and 36,055 deaths in 2019 alone, reflecting an 85.5% increase in global DENV incidence between 1990 and 2019, highlighting the escalating burden of DENV-related illnesses worldwide (Yang et al., 2021). Particularly endemic in South-East Asia and South Asia, dengue presents significant social, economic, and healthcare challenges in these regions (Shepard et al., 2016). Statistical models predict an expansion of DENV transmission geographically due to ongoing climate change and urbanization, with over 6 billion people projected to be at risk of DENV infection by 2080 (Messina et al., 2019). The exponential increase in DENV infections over the past few decades has made the search for a dengue vaccine a critical priority. Significant progress has been made in recent years in vaccine development; however, the long-term efficacy and safety of dengue vaccines in regions where the disease is endemic remain uncertain (Screaton et al., 2015; Thomas, 2023). YFV primarily afflicts tropical and subtropical regions of Africa, South, and Central America. In 2018, an estimated 109,000 severe infections and 51,000 deaths were attributed to yellow fever in these areas (Gaythorpe et al., 2021). Despite the availability of effective vaccines, YFV has sparked multiple pandemics and resurged as a major global health threat (Lindsey et al., 2022). YFV is mainly transmitted by the anthropophilic *Aedes* mosquitoes, which are prevalent in tropical and subtropical regions, but historically YFV is absent from the Asia-Pacific region. Increasing exchanges between Africa and Asia have led to imported YFV cases in non-endemic areas, posing a new viral threat to Asia (Wasserman et al., 2016). Asian-Pacific *Aedes* mosquitoes are competent vectors for YFV, with a higher potential to transmit the virus and pose a greater risk of transmission to human populations compared to *Aedes aegypti* from YFV-endemic regions in Africa (de Guilhem de Lataillade et al., 2020). The growing global interconnectedness facilitates YFV spread into low-risk or previously YFV-free regions, emphasizing the necessity of ongoing surveillance (Reno et al., 2020). Zika virus (ZIKV) has rapidly emerged since 2007, instigating epidemics across Micronesia, the South Pacific, and the Americas (Weaver et al., 2016; Musso et al., 2019). Designated as a Public Health Emergency of International Concern (PHEIC) by the World Health Organization (WHO) on February 1, 2016 (Gulland, 2016), the most recent outbreak in Brazil saw an estimated 440,000 to 1,300,000 cases of ZIKV infection reported (Bogoch et al., 2016). However, global mortality data for ZIKV is relatively limited compared to other viral infections, as most ZIKV infections are asymptomatic or result in mild symptoms such as fever, rash, joint pain, and conjunctivitis (Wikan and Smith, 2016). Notably, the outbreak in Brazil raised significant concerns due to the dramatic increase in cases of microcephaly (Wikan and Smith, 2016). The mortality rate was 52.6 deaths per 1,000 person-years among live-born children with congenital Zika syndrome, compared to 5.6 deaths per 1,000 person-years among those without the syndrome (Paixao Enny et al., 2022). ZIKV infections are predominantly concentrated in Latin American and Caribbean nations, with sporadic cases elsewhere (Guo et al., 2022). Studies predict a heightened risk of ZIKV transmission in forthcoming climate scenarios, particularly in regions like southern and Eastern Europe, northern America, and temperate areas of Asia such as northern China and southern Japan (Blagrove et al., 1930). The number of new people at risk of ZIKV infection is projected to exceed 1.3 billion by 2050 due to warming temperatures (Ryan et al., 2021). The rapid spread of flaviviruses, both locally and globally, is facilitated by various eco-epidemiological factors, including global warming, urban development, and increased intercontinental travel (Baker et al., 2022). Consequently, flaviviruses are recognized as potential candidates for future viral pandemics (Pierson and Diamond, 2020).

The emergence of the COVID-19 pandemic reportedly triggered changes in the epidemiological patterns of various infectious diseases (Xiao et al., 2021; Hartner et al., 2024). Nonpharmaceutical interventions such as lockdowns, quarantine, universal masking, and physical distancing measures aimed at combating COVID-19 were estimated to have averted approximately 0.72 million dengue cases that would have otherwise occurred in 2020 across Latin America and Southeast Asia (Chen et al., 2022). Nevertheless, disruptions caused by COVID-19 have impeded public access to healthcare services, leading to a dual burden of COVID-19 and dengue (Harapan et al., 2021; Luo et al., 2024). Moreover, the efforts to combat SARS-CoV-2 came at the expense of flavivirus diagnosis and control practices, leading to the simultaneous circulation of SARS-CoV-2 and flavivirus in Brazil (da Silva et al., 2021). The disruption of immunization and drug administration campaigns during the COVID-19 era has left numerous children at risk of yellow fever and other neglected tropical diseases (Jafari et al., 2021). This complex interplay necessitates a thorough examination of potential shifts in the burden of flavivirus infections before and during the COVID-19 pandemic. Furthermore, robust assessments of flavivirus incidence and forecasts of future trends are indispensable for effective intervention planning to mitigate the risk of significant outbreaks.

## 2 Methods

### 2.1 Data source and data collection

The Global Burden of Diseases, Injuries, and Risk Factors Study (GBD) is a collaborative international effort led by the Institute for Health Metrics and Evaluation (IHME) and involving over 11,000 contributors (Ferrari et al., 2024). Its primary focus lies in estimating global population demographics, fertility rates, morbidity, and mortality. This cross-sectional study utilized annual estimations of region-, country-, and age-specific incidence numbers and crude rates of three prevalent flavivirus infections (Dengue, Zika, and yellow fever) obtained from the GBD 2021 through the Global Health Data Exchange (GHDx) query tool^1^ (Ferrari et al., 2024). The study spanned individuals of all age groups across 204 countries and territories from 2011 to 2021, categorizing the population into twenty age brackets of five years each: <5, 5–9, 10–14, 15–19, 20–24, 25–29, 30–34, 35–39, 40–44, 45–49, 50–54, 55–59, 60–64, 65–69, 70–74, 75–79, 80–84, 85–89, 90–94, and >95 years of age. Furthermore, the 204 countries and territories are organized into 21 GBD regions based on epidemiological similarities and geographic proximity (Ferrari et al., 2024). All participants met the inclusion criteria set forth by the GBD Study. The study protocol received approval from the University of Washington’s research ethics board and will be conducted in strict adherence to the university’s policies and procedures, as well as compliance with relevant federal, state, and local laws (Ferrari et al., 2024).

### 2.2 Case definition

In the GBD 2021, all cases of dengue fever, including classical dengue [defined by the International Classification of Diseases version 10 (ICD-10) code under heading A90], and dengue hemorrhagic fever (ICD-10 code under heading A91) are accounted for. yellow fever cases are identified by any ICD-10 code under heading A95, while Zika virus (ZIKV) infections are defined by any ICD-10 code between U06 and U06.9 (Ferrari et al., 2024). A confirmed case of dengue is identified through one or more of the following methods, in accordance with the World Health Organization’s criteria: isolation of the dengue virus in a cell culture; identification of the virus’s genetic material via polymerase chain reaction (PCR); detection of the non-structural protein 1 (NS1) antigen using enzyme-linked immunosorbent assay (ELISA) or a rapid diagnostic test; and serological identification of immunoglobulin M (IgM) or immunoglobulin G (IgG) antibodies through ELISA, rapid tests, or hemagglutination inhibition assays. A diagnosis of yellow fever can be established based on the following criteria: (i) detection of yellow fever M (IgM) antibodies in a patient not vaccinated against yellow fever within 30 days prior to the onset of symptoms; or (ii) positive liver histopathology findings from a postmortem examination; or (iii) a clear epidemiological connection to a confirmed case or an outbreak, as per the World Health Organization’s guidelines. Additionally, the diagnosis can be supported by either: (a) no yellow fever vaccination within 30 days before the illness began, coupled with one of the following: (i) identification of yellow fever-specific IgM antibodies; or (ii) a fourfold or greater increase in yellow fever IgM or IgG antibody levels between serum samples taken during the acute phase and the recovery phase, or both; or (iii) identification of yellow fever-specific neutralizing antibodies; or (b) no yellow fever vaccination within 14 days before the onset of symptoms, and one of the following: (i) detection of the yellow fever virus genome in blood or other organs through polymerase chain reaction (PCR); or (ii) detection of yellow fever antigen in blood, liver, or other organs via immunoassay; or (iii) isolation of the yellow fever virus. A patient is considered to have a confirmed case of recent Zika virus infection if they fulfill the criteria for a suspected case and also have laboratory evidence supporting this, which includes: (i) the presence of Zika virus RNA or antigen detected in any biological sample (such as serum, urine, saliva, tissue, or whole blood); OR (ii) a positive test for Zika virus-specific IgM antibodies along with a Zika virus neutralizing antibody titer (PRNT90) that is equal to or greater than 20 and is fourfold or greater in comparison to the titers against other flaviviruses, along with the exclusion of other flavivirus infections; OR (iii) in the case of postmortem specimens, identification of the Zika virus genome in fresh or paraffin-embedded tissue using molecular techniques, or identification through immunohistochemistry, according to the Pan American Health Organization’s 2016 guidelines (Ferrari et al., 2024).

### 2.3 Global and national socioeconomic status

The socio-demographic index (SDI) is a composite indicator of background social and economic conditions that influence health outcomes in each location (Ferrari et al., 2024). This composite indicator encompasses three key indices: (1) total fertility rate for individuals under 25 years old; (2) mean education level among those aged 15 years and above; and (3) lag-distributed income *per capita*. The composite SDI is derived by standardizing these three indices for a specific location-year to yield the geometric mean. Based on the resulting SDI score, regions and countries are categorized into five distinct quintiles: low SDI (0–0.455), low-middle SDI (0.455–0.608), middle SDI (0.608–0.690), high-middle SDI (0.690–0.805), and high SDI (0.805–1). Additionally, the World Bank categorizes economies worldwide into four income tiers: low (<$1,045), lower-middle ($1,046 to $4,095), upper-middle ($4,096 to $12,695), and high-income (>$12,695) (World Bank Blogs, 2021). These classifications are determined by the gross national income *per capita* in current USD from 2020, utilizing the Atlas method exchange rates.

### 2.4 Statistical analysis

This study calculated the age-standardized incidence rate (ASIR) of flavivirus infections per 100,000 population employing the following formula:


$$\mathrm{ASIR}=\frac{\sum_{i=1}^{A}a_{i}w_{i}}{\sum_{i=1}^{A}w_{i}}\times 100,000$$

In this context, $a_{i}$ signifies the age-specific incidence rate within the ith age subgroup, and $w_{i}$ indicates the population count of individuals within the same age category sourced from the GBD Study Population Estimates 1950–2021 (Schumacher et al., 2024). To gauge the temporal trends in flavivirus infection burden, we computed the estimated annual percentage changes (EAPCs) in ASIR. This involved fitting a regression line to the natural logarithm of the rates, represented as y=α+βx+ε, where y=ln(ASR) and x=calendar year. The EAPC was derived as 100×(exp(β)−1), with corresponding 95% confidence interval (CI) obtained from the linear regression model (Gao et al., 2012). Additionally, we conducted Pearson correlation analysis to assess the association between ASIR and SDI quintile and visualized the results with locally weighted scatterplot smoothing (LOWESS) curves. All statistical analyses and mapping were performed using R software, version 4.1.0 (R Foundation for Statistical Computing), with significance set at *p* < 0.05.

## 3 Results

### 3.1 Global incidence of DENV, YFV, and ZIKV infections

In 2021, there were an estimated 59,220,428 flavivirus infections reported worldwide, with 27,480,266 incidents among males and 31,740,162 among females, marking a 3.5% increase from 2019 (Supplementary Table S1). According to the WHO, global yellow fever vaccination coverage, defined as the proportion of the target population receiving one dose of the vaccine in a given year, was 47% in 2021 (WHO, 2024). Notably, vaccination coverage in high-burden regions has declined from 2019 to 2021, with Africa seeing a drop from 47 to 45% and the Americas from 61 to 58%. In 2021, there were 290,766 cases and 4,602 deaths attributable to DENV and YFV infections, respectively, corresponding to 0.38 and 0.06 per 100,000 people. However, deaths related to ZIKV infection are rare and nearly negligible. The global ASIR of flavivirus infections stood at 715.69 per 100,000 population in 2021. Notably, the ASIR experienced an average annual increase of 5.08% (95% CI 4.12 to 6.05) from 2011 to 2016, followed by a decrease of −8.37% (95% CI −12.46 to −4.08) per year from 2016 to 2019. Furthermore, the ASIR remained stable during 2019–2021, with an average annual change of 0.69% (95% CI −0.96 to 2.37) globally (Supplementary Table S1).

The number of incident cases of DENV infection rose by 3.8%, from 56,799,358 in 2019 to 58,964,185 in 2021 (Table 1). Nevertheless, the number of both YFV and ZIKV infections declined between 2019 and 2021, with totals of 86,509 and 169,734 cases, respectively, in 2021 (Table 1). From 2011 to 2021, the temporal trends in the ASIR of DENV infection is consistent with those of the combined ASIR for the three prevalent flavivirus infections. However, the ASIR of YFV infection showed a declining trend over the same period. Additionally, a significant increase in the ASIR of ZIKV infection occurred during 2011–2016 [with an average annual change of 157.59% (95% CI 100.15 to 231.52)], while globally, the ASIR of ZIKV infection decreased by an annual average of −66.1% (95% CI −79.29 to −44.49) from 2016 to 2019, and −30.53% (95% CI −30.56 to −30.49) from 2019 to 2021 (Table 1).

**Table 1:** The number and age-standardized incidence rates (ASIR, per 100,000) of DENV, YFV, and ZIKV infections in 2021, as well as the temporal trends.

Characteristics	2021		2011–2016		2016–2019		2019–2021	
	Case number	ASIR per 100,000	Percent change (%)	EAPC (95% CI)	Percent change (%)	EAPC (95% CI)	Percent change (%)	EAPC (95% CI)
**Dengue**								
**Overall**	58,964,185	752.04	20.3	2.8 (1.53 to 4.08)	−10.2	−4.68 (−5.7 to −3.64)	3.8	0.92 (−0.86 to 2.73)
*Sex*								
Male	27,346,972	694.78	19.6	2.7 (1.48 to 3.95)	−9.7	−4.55 (−5.54 to −3.55)	3.6	0.83 (−0.88 to 2.56)
Female	31,617,213	810.65	20.8	2.87 (1.57 to 4.18)	−10.6	−4.79 (−5.85 to −3.72)	4	1 (−0.85 to 2.87)
*World Bank classification*								
High-income	685,622	56.35	13.3	1.97 (0.48 to 3.49)	−24	−9.51 (−11.05 to −7.94)	4	1.38 (0.88 to 1.89)
Upper-middle-income	17,444,892	724.12	29.1	4.98 (2.11 to 7.92)	−33.6	−13.75 (−15.7 to −11.76)	2.9	1.04 (−0.34 to 2.43)
Lower-middle-income	40,316,675	1185.11	14.9	1.18 (0.98 to 1.38)	6.3	0.54 (0.46 to 0.62)	4.2	0.77 (−1.19 to 2.76)
Low-income	496,614	70.35	18.1	0.89 (0.83 to 0.94)	9.5	0.65 (0.35 to 0.94)	1.5	−1.62 (−2.28 to −0.96)
*GBD regions*								
High-income Asia Pacific	485,712	294.01	30.7	5.8 (3.2 to 8.45)	−30.5	−11.59 (−13.3 to −9.84)	4.3	2.37 (1.05 to 3.71)
Central Asia	0	0	NA	NA	NA	NA	NA	NA
East Asia	61,440	4.27	7.8	0.63 (0.48 to 0.78)	6.8	1.45 (1.26 to 1.63)	4.1	1.47 (−2.93 to 6.06)
South Asia	31,812,189	1726.94	15.1	1.36 (1.16 to 1.56)	5.7	0.56 (0.51 to 0.61)	3.2	0.52 (−1.48 to 2.56)
Southeast Asia	6,728,444	971.89	31.3	4.71 (3.03 to 6.41)	−3.5	−2.35 (−3.29 to −1.41)	6.5	2.18 (0.3 to 4.09)
Australasia	18,448	58.99	−11.5	−4.05 (−4.27 to −3.84)	−8.3	−4.72 (−5.71 to −3.72)	6.7	1.41 (−1.65 to 4.56)
Caribbean	227,073	475.93	−34.2	−9.34 (−10.79 to −7.87)	−3.9	−2.05 (−2.61 to −1.49)	0.6	−0.38 (−2.29 to 1.57)
Central Europe	0	0	NA	NA	NA	NA	NA	NA
Eastern Europe	0	0	NA	NA	NA	NA	NA	NA
Western Europe	0	0	NA	NA	NA	NA	NA	NA
Andean Latin America	391,708	593.22	21.7	2.37 (1.29 to 3.46)	−3.3	−3.07 (−3.67 to −2.48)	9.6	2.97 (−1.01 to 7.1)
Central Latin America	2,886,641	1140.37	−10.3	−3.06 (−3.9 to −2.21)	−40	−16.66 (−18.87 to −14.39)	4.1	1.56 (1.03 to 2.1)
Southern Latin America	80,129	118.83	55.6	8.61 (5.51 to 11.8)	−5.5	−2.8 (−3.08 to −2.52)	0.2	−0.59 (−0.82 to −0.37)
Tropical Latin America	13,043,195	5774.82	39.2	6.41 (3.07 to 9.86)	−32.5	−13.42 (−15.26 to −11.54)	2.7	0.48 (−0.7 to 1.67)
North Africa and Middle East	53,391	8.5	−39.1	−12.01 (−14.5 to −9.44)	3.1	−0.54 (−0.58 to −0.49)	17.7	7.09 (−4.09 to 19.56)
High-income North America	1,376	0.36	10.3	1.07 (0.71 to 1.43)	2.9	0.13 (−0.26 to 0.53)	16.4	7.25 (−5.2 to 21.33)
Oceania	63,970	486.03	16.4	0.44 (0.05 to 0.84)	18.3	3.26 (2.76 to 3.76)	2.3	−1.16 (−7.49 to 5.61)
Central Sub-Saharan Africa	245,097	178.08	19.2	0.43 (0.34 to 0.52)	12.7	1.14 (0.67 to 1.61)	1.2	−2.02 (−3.03 to −1)
Eastern Sub-Saharan Africa	387,627	94.37	15.9	0.49 (0.23 to 0.75)	3.8	−1 (−1.11 to −0.9)	3.9	−0.17 (−0.75 to 0.41)
Southern Sub-Saharan Africa	1,087	1.32	−4	−2.04 (−2.83 to −1.23)	7.4	1.43 (1.2 to 1.66)	15.2	6.53 (0.86 to 12.51)
Western Sub-Saharan Africa	2,476,656	512.53	17.2	0.04 (−0.1 to 0.18)	12.9	1.09 (1.02 to 1.15)	10.1	2.07 (−3.07 to 7.47)
**Yellow fever**								
**Overall**	86,509	1.15	−30.7	−7.5 (−13.8 to −0.75)	−30.2	−11.13 (−14.84 to −7.26)	−5.5	−3.37 (−5.45 to −1.24)
*Sex*								
Male	60,460	1.57	−30.9	−7.6 (−13.82 to −0.92)	−30.1	−11.11 (−14.77 to −7.3)	−5.6	−3.43 (−5.59 to −1.22)
Female	26,048	0.70	−30.2	−7.3 (−13.76 to −0.37)	−30.4	−11.17 (−15 to −7.17)	−5.2	−3.25 (−5.15 to −1.32)
*World Bank classification*								
High-income	29	0	−23.6	−5.47 (−5.79 to −5.15)	−11.9	−4.37 (−5.05 to −3.68)	−4.6	−2.72 (−5.22 to −0.15)
Upper-middle-income	2,359	0.1	−21.1	−4.67 (−5.54 to −3.8)	−11.3	−0.41 (−60.29 to 149.78)	−5.1	−2.88 (−4.67 to −1.05)
Lower-middle-income	37,149	1.04	6.4	−2.11 (−9.44 to 5.81)	−43.9	−17.55 (−27.48 to −6.26)	−7.7	−4.79 (−6.5 to −3.06)
Low-income	46,943	6.42	−52.2	−13.75 (−19.9 to −7.13)	−13.8	−7.13 (−7.29 to −6.97)	−3.7	−4.25 (−6.56 to −1.89)
*GBD regions*								
High-income Asia Pacific	0	0	NA	NA	NA	NA	NA	NA
Central Asia	0	0	NA	NA	NA	NA	NA	NA
East Asia	0	0	NA	NA	NA	NA	NA	NA
South Asia	0	0	NA	NA	NA	NA	NA	NA
Southeast Asia	0	0	NA	NA	NA	NA	NA	NA
Australasia	0	0	NA	NA	NA	NA	NA	NA
Caribbean	38	0.08	−25.1	−6.09 (−6.37 to −5.81)	−12.6	−10.32 (−33.46 to 20.87)	−9.5	−5.09 (−9.47 to −0.51)
Central Europe	0	0	NA	NA	NA	NA	NA	NA
Eastern Europe	0	0	NA	NA	NA	NA	NA	NA
Western Europe	0	0	NA	NA	NA	NA	NA	NA
Andean Latin America	1,090	1.64	−19.3	−5.27 (−6.62 to −3.91)	−9.5	−6.08 (−10.7 to −1.22)	−3.3	−3.33 (−5.36 to −1.26)
Central Latin America	178	0.07	−19.1	−5.2 (−5.63 to −4.77)	−15	−6.45 (−10.37 to −2.36)	−9.6	−5.28 (−8.16 to −2.31)
Southern Latin America	709	1.07	−21.4	−5.2 (−5.93 to −4.46)	−10.8	−3.71 (−8.62 to 1.46)	−5.5	−3.43 (−5.15 to −1.68)
Tropical Latin America	187	0.09	−23.2	−5.52 (−6.21 to −4.83)	−16.1	−1.05 (−90.15 to 893.71)	−6.9	−3.93 (−4.68 to −3.16)
North Africa and Middle East	6,659	1.04	−31.3	−8.67 (−8.96 to −8.38)	−17.3	−7.65 (−7.97 to −7.32)	−5.8	−4.06 (−7.39 to −0.61)
High-income North America	0	0	NA	NA	NA	NA	NA	NA
Oceania	0	0	NA	NA	NA	NA	NA	NA
Central Sub-Saharan Africa	10,243	7.12	58.4	1.29 (−17.06 to 23.71)	−71	−33.12 (−53.41 to −3.98)	−6.2	−5.69 (−7.69 to −3.65)
Eastern Sub-Saharan Africa	24,416	5.41	−28.5	−8.65 (−9.74 to −7.54)	−14.1	−7.16 (−7.38 to −6.95)	−4.2	−4.5 (−7.17 to −1.75)
Southern Sub-Saharan Africa	0	0	NA	NA	NA	NA	NA	NA
Western Sub-Saharan Africa	42,988	8.13	−51.4	−14.16 (−20.67 to −7.11)	−13.1	−7.69 (−9.16 to −6.2)	−6	−5.85 (−7.36 to −4.33)
**ZIKV infection**								
**Overall**	169,734	2.13	24,856	157.59 (100.15 to 231.52)	−96.3	−66.1 (−79.29 to −44.49)	−50.8	−30.53 (−30.56 to −30.49)
*Sex*								
Male	72,833	1.83	25,702	158.77 (100.84 to 233.39)	−96.2	−66.04 (−79.03 to −45.02)	−50.8	−30.53 (−30.58 to −30.49)
Female	96,900	2.46	24,264	156.69 (99.6 to 230.11)	−96.3	−66.14 (−79.48 to −44.13)	−50.9	−30.53 (−30.56 to −30.5)
*World Bank classification*								
High-income	1,066	0.09	501,179	314.93 (134.45 to 634.34)	−99.7	−80.45 (−93.67 to −39.62)	−51	−30.15 (−30.22 to −30.07)
Upper-middle-income	153,753	6.24	48,249	195.54 (129.14 to 281.19)	−95.9	−65.43 (−79.38 to −42.04)	−50.8	−29.89 (−29.91 to −29.88)
Lower-middle-income	14,697	0.44	4,658	89.95 (59.69 to 125.94)	−97	−68.49 (−83.87 to −38.46)	−51	−31.01 (−31.02 to −30.99)
Low-income	0	0	NA	NA	NA	NA	NA	NA
*GBD regions*								
High-income Asia Pacific	0	0	NA	NA	NA	NA	NA	NA
Central Asia	0	0	NA	NA	NA	NA	NA	NA
East Asia	0	0	NA	NA	NA	NA	NA	NA
South Asia	0	0	NA	NA	NA	NA	NA	NA
Southeast Asia	0	0	NA	NA	NA	NA	NA	NA
Australasia	0	0	NA	NA	NA	NA	NA	NA
Caribbean	6,432	13.01	404,579	298.81 (124.68 to 607.9)	−99	−75.89 (−85.34 to −60.35)	−51.2	−30.69 (−30.7 to −30.68)
Central Europe	0	0	NA	NA	NA	NA	NA	NA
Eastern Europe	0	0	NA	NA	NA	NA	NA	NA
Western Europe	0	0	NA	NA	NA	NA	NA	NA
Andean Latin America	29,726	44.61	164,120	249.97 (133.4 to 424.78)	−81.5	−53.34 (−77.98 to −1.12)	−50	−30.55 (−30.56 to −30.53)
Central Latin America	51,041	19.99	18,660	141.68 (85.45 to 214.95)	−97.9	−69.15 (−86.19 to −31.09)	−51.1	−30.4 (−30.45 to −30.35)
Southern Latin America	107	0.16	1,481,650	384.54 (130.44 to 918.82)	−99.8	−86.32 (−90.34 to −80.65)	−51.4	−30.65 (−30.72 to −30.59)
Tropical Latin America	82,426	35.51	25100.4	167.35 (123.47 to 219.86)	−93.3	−59.22 (−83.96 to 3.69)	−50.9	−30.52 (−30.54 to −30.5)
North Africa and Middle East	0	0	NA	NA	NA	NA	NA	NA
High-income North America	1	0	1,232,261	373.1 (132.48–862.77)	NA	NA	NA	NA
Oceania	0	0	65,842	178.27 (59.38–385.83)	NA	NA	NA	NA
Central Sub-Saharan Africa	0	0	NA	NA	NA	NA	NA	NA
Eastern Sub-Saharan Africa	0	0	NA	NA	NA	NA	NA	NA
Southern Sub-Saharan Africa	0	0	NA	NA	NA	NA	NA	NA
Western Sub-Saharan Africa	0	0	NA	NA	NA	NA	NA	NA

NA: Not applicable.

**Figure 1:**
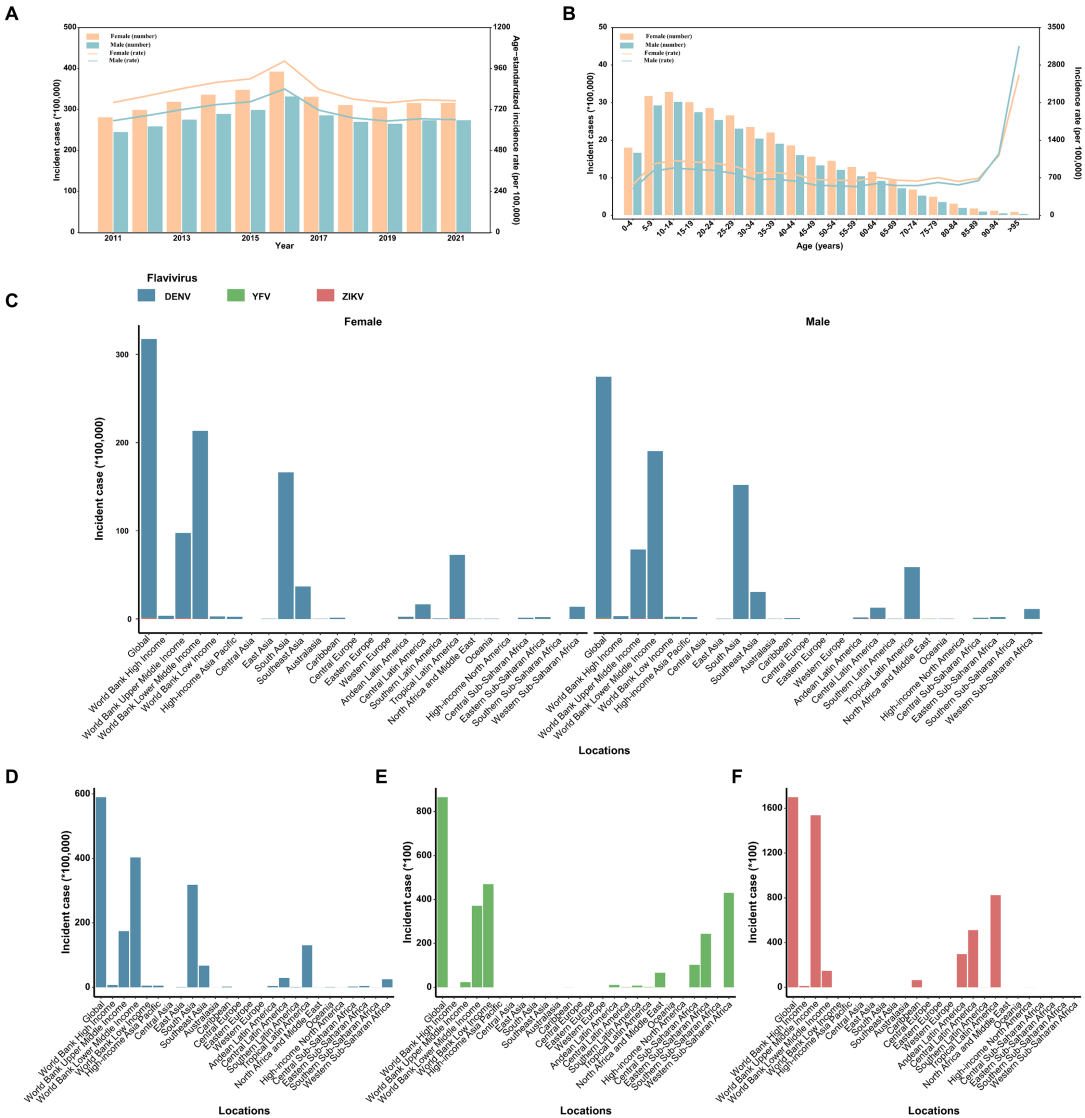
The distribution and trend of the number and age-standardized rate of incidence for three prevalent flavivirus (DENV, YFV, and ZIKV) infections, by sex. **(A)** The number and age-standardized rate of incidence from 2011 to 2021. **(B)** The number and rate of incidence in 2021 across age groups. **(C)** The distribution of incident cases across the globe, in different World Bank income classification and 21 GBD regions, 2021. **(D)** The distribution of incident cases of dengue across the globe, in different World Bank income classification and 21 GBD regions, 2021. **(E)** The distribution of incident cases of yellow fever across the globe, in different World Bank income classification and 21 GBD regions, 2021. **(F)** The distribution of incident cases of ZIKV infection across the globe, in different World Bank income classification and 21 GBD regions, 2021. DENV, Dengue virus; YFV, yellow fever virus; ZIKV, Zika virus.

**Figure 2:**
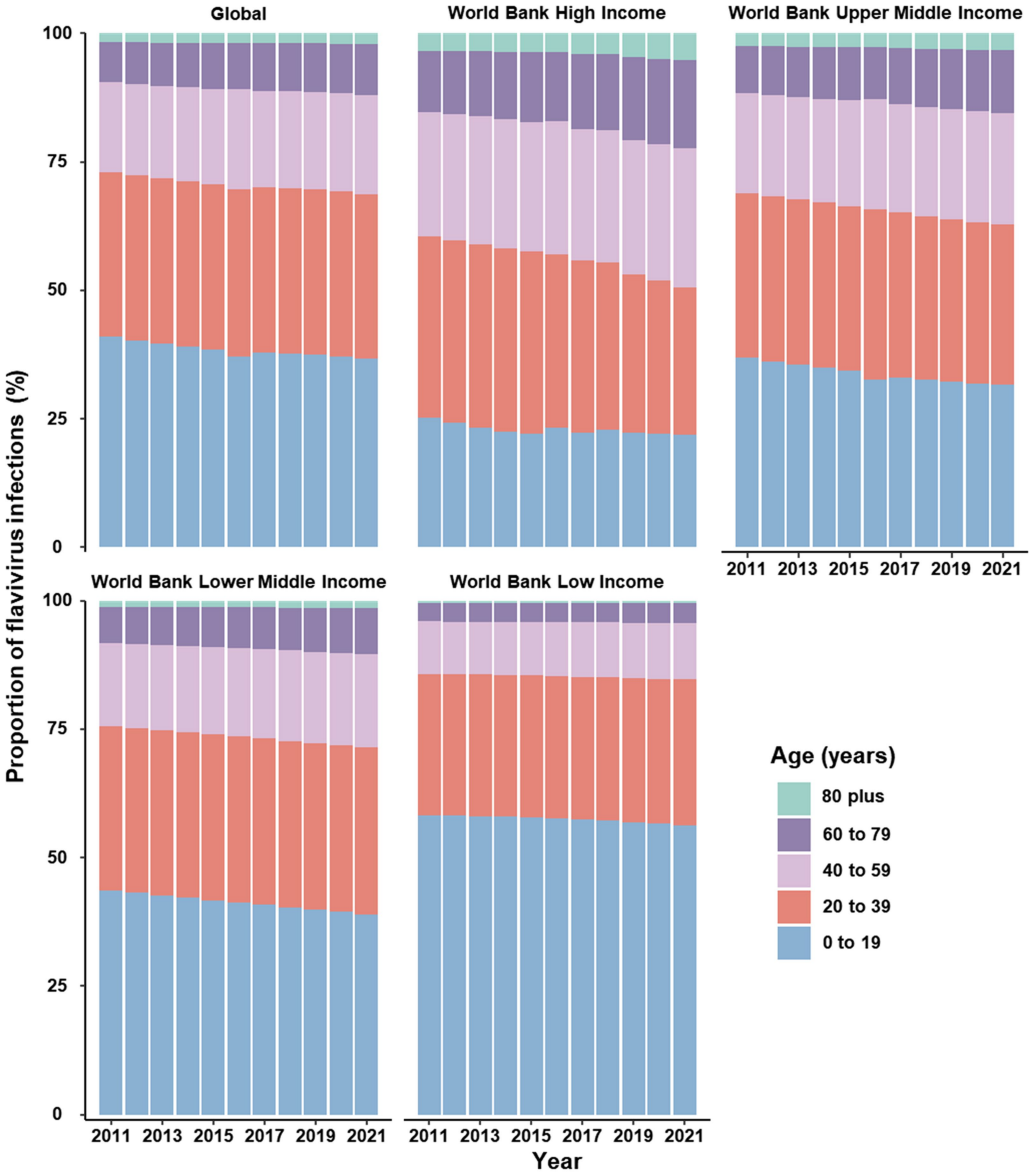
Proportion of cases infected with flavivirus stratified by age groups, worldwide and across four income levels, 2011–2021.

Between 2011 and 2021, globally, the number of incident infection and ASIR were consistently higher among females compared to males (Figure 1A). In terms of specific flavivirus infections, the ASIR of DENV infection was slightly higher in females versus males among individuals under 95 years old (Supplementary Figure S1A). The ASIR of YFV infection was generally more frequent among males than females across all age groups, with the gap decreasing with increasing age (Supplementary Figure S1B). In the case of ZIKV infection, females under 70 years old had a higher ASIR than males of the same age, and this trend reversed after the age of 70 (Supplementary Figure S1C). The vast majority of incident cases worldwide were attributed to DENV infection (Figure 1C; Supplementary Figure S2). By age, the incidence of flavivirus infections peaked in the oldest age group among both sexes, even though the age group of 10–14 years had the highest number of cases (Figure 1B). Notably, the proportion of infected cases aged 60 years and above increased over the years globally, especially in higher-income regions (Figure 2 and Supplementary Table S2).

### 3.2 Incidence burden across four income-classified regions

In 2021, the incidence for flavivirus infections were highest in the region with lower-middle income (Supplementary Figure S3 and Supplementary Table S1). Between 2011 and 2016, the high-income region had the fastest increase in the ASIR [average annual change 10.65% (95% CI 2.61 to 19.32)], and a decrease in the ASIR was found only in low-income region [average change −1.79% per year (95% CI −3.28 to −0.28)]. Moreover, all income-classified regions showed a decreasing trend in the ASIR from 2016 to 2019 and then remained stable from 2019 to 2021 (Supplementary Table S1). The regions with the highest incidence of DENV, YFV, and ZIKV infection were the low-middle income region, the low-income region, and the upper-middle income region, respectively (Supplementary Figures S4, S5). Notably, in the 2016 global ZIKV epidemic, more than 80% of reported ZIKV cases originated from the upper-middle income region (Supplementary Figure S4C).

### 3.3 Incidence burden across 21 GBD regions

In 2021, South Asia documented the most incident cases of flavivirus infections, totaling 31,812,189, whereas Tropical Latin America had the highest ASIR for flavivirus infections, standing at 5464.54 per 100,000 population (Supplementary Table S1). Despite South Asia’s high population and vulnerability to mosquito-borne infections, there were no recorded YFV or ZIKV infections in the region in 2021 (Table 1). Notably, no cases of DENV, YFV, or ZIKV infections were recorded in Central, Eastern, or Western Europe, nor in Central Asia, for either sex (Figure 1C). Between 2011 and 2016, the largest increases in ASIR of flavivirus infections occurred in the regions of Latin America (Southern, Andean, and Tropical) and High-income North America (Supplementary Table S1). From 2016 to 2019, the largest increases in ASIR of flavivirus infections were found in Oceania [2.6% (2.4 to 2.8)], East Asia [1.79% (1.6 to 1.97)], and Southern Sub-Saharan Africa [1.56% (1.32 to 1.81); Supplementary Table S1]. The most significant fluctuation in the ASIR for flavivirus infections occurred in High-income North America and Southern Sub-Saharan Africa during 2019 to 2021 (Supplementary Table S1). The Americas and the Caribbean witnessed the highest incidence of flavivirus infections during 2015–2017. Moreover, the regions of Australasia and North Africa and Middle East experienced a significant decrease in the incidence of flavivirus infections (Supplementary Figure S6).

### 3.4 Incidence burden at the country level

**Figure 3:**
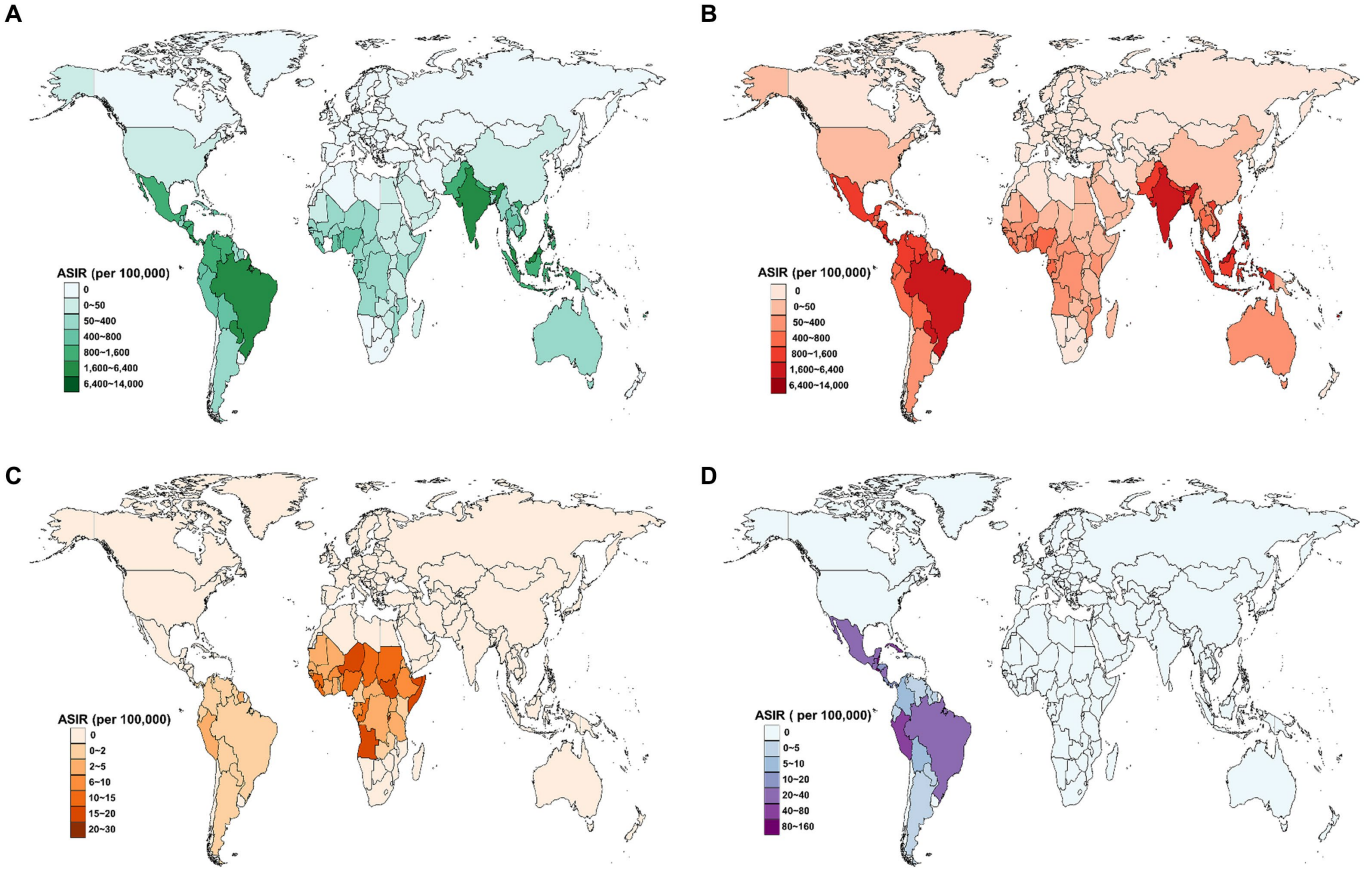
Estimated age-standardized incidence rate (ASIR) for three prevalent flavivirus infections in 2021, by country. **(A)** The combined ASIR of DENV, YFV, and ZIKV infections. **(B)** The ASIR of DENV infection. **(C)** The ASIR of YFV infection. **(D)** The ASIR of ZIKV infection. DENV, Dengue virus; YFV, yellow fever virus; ZIKV, Zika virus.

In 2021, the ASIR for flavivirus infections was highest in Tonga (13388.9 per 100,000 population), followed by Seychelles (11565.2 per 100,000 population), Comoros (11075.1 per 100,000 population), and Marshall Islands (9646.5 per 100,000 population; Figure 3A). Meanwhile, 80 countries and territories reported no documented cases of DENV, YFV, or ZIKV infections. The global distribution of flavivirus infections has been predominantly shaped by the epidemic patterns of DENV infection (Figure 3B). Worldwide, YFV infection has been reported in only 47 countries and territories, with Burundi having the highest ASIR of 29.1 per 100,000 population (Figure 3C). In comparison, ZIKV infection affected 34 countries and territories, all within the American region (Figure 3D). The top three countries with the highest ASIR of ZIKV infection in 2021 were El Salvador (153.5 per 100,000), Belize (103 per 100,000), and Peru (79.1 per 100,000).

**Figure 4:**
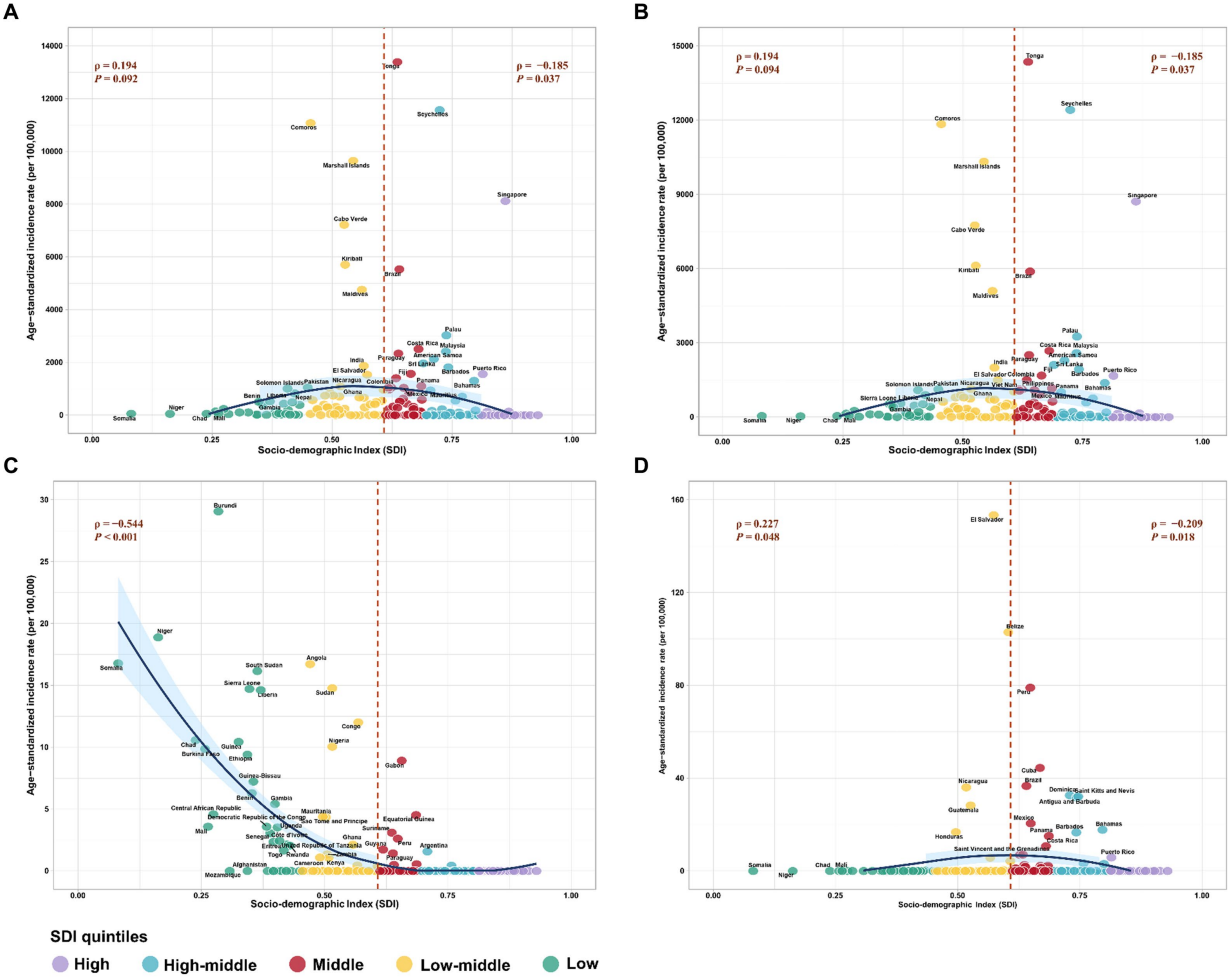
The correlation between age-standardized incidence rate (ASIR) of three prevalent flavivirus infections and socio-demographic index (SDI). **(A)** The combined ASIR of DENV, YFV, and ZIKV infections versus SDI. **(B)** The ASIR of DENV infection versus SDI. **(C)** The ASIR of YFV infection versus SDI. **(D)** The ASIR of ZIKV infection versus SDI. The different colors stand for different SDI quintiles. The *ρ* indices and *p*-values were derived from Pearson correlation analysis. DENV, Dengue virus; YFV, yellow fever virus; ZIKV, Zika virus.

Additionally, the correlation between the ASIR of flavivirus infection and the SDI level exhibited an inverted “U” shape, peaking at SDI values around 0.6 and then declining as SDI values increased (Figure 4A). The patterns of the ASIR of DENV infection or ZIKV infection versus the SDI value were similar to those of the combined ASIR of the three flavivirus infections (Figures 4B,D). Furthermore, the ASIR of YFV infection decreased exponentially with increases in SDI level (Figure 4C).

## 4 Discussion

To the best of our knowledge, this is the first study to evaluate the incidence and trends of the three common flavivirus infections—DENV, YFV, and ZIKV—at global, regional, and national levels. To help inform the optimal implementation of public health interventions, robust estimates of flavivirus incidence and trends of future dynamics are essential (Gaythorpe et al., 2021). In this study, we provided the most up-to-date estimates of flavivirus infection incidence across the globe from 2011 to 2021. In 2021, nearly 60 million flavivirus infections were estimated to have occurred in 124 countries and territories, marking a 1.12-fold increase from 2011. Globally, the incidence of flavivirus infections peaked in 2016, which may be attributed to the 2015–2016 El Niño climate phenomenon (Anyamba et al., 2019). A significant overlap between the El Niño phenomenon, regional climate anomalies, and hyperendemic for DENV in South America and Southeast Asia have been demonstrated by previous studies (Anyamba et al., 2019; Ferreira et al., 2022; Liyanage et al., 2022). Additionally, the unique climatic conditions caused by the El Niño event were optimal for the transmission of ZIKV in the regions of America (Paz and Semenza, 2016; Caminade et al., 2017; Anyamba et al., 2019). The geographical distribution of flaviviruses has the potential to expand further, as their primary vectors are predicted to spread into temperate regions (Kraemer et al., 2019). The trajectory of ZIKV’s spread in the Western Hemisphere illustrates the introduction of a previously obscure vector-borne disease into new ecological systems and populations, leading to swift dissemination with significant implications for human health (Lazear and Diamond, 2016). Therefore, timely surveillance to detect changes in pathogen distribution is essential for providing early warnings to public health officials to implement interventions, as evidenced by the global COVID-19 pandemic (Dong et al., 2020).

Notably, Pierson and Diamond (2020) indicated that flaviviruses are now globally distributed, infecting up to 400 million people annually, a figure significantly higher than the GBD estimation. This discrepancy could be attributed to several factors, including variations in data sources and modeling methodologies. Pierson and Diamond (2020) investigation included the presence of flavivirus infections derived from both peer-reviewed literature and HealthMap alerts. The GBD used cases of flavivirus infections reported by countries to the WHO and other global monitoring entities (Ferrari et al., 2024). This likely led to an underestimation of flavivirus infections due to under-reporting caused by limited health system capacity or misdiagnosis, even in many hyperendemic countries (Kakkar, 2012; Petersen et al., 2016; Shearer et al., 2018). Most flaviviruses are known to cause subclinical infections that are typically undetectable by existing clinical-based disease surveillance programs. For example, it was estimated that only 96 million of the 390 million global dengue cases in 2010 manifest apparent sign or symptom (Bhatt et al., 2013). Additionally, approximately 20% of individuals infected with ZIKV develop a clinically apparent febrile illness (Lazear and Diamond, 2016). More than 85% of YFV infection cases were either asymptomatic or presented with mild illness (Ndeffo-Mbah and Pandey, 2020). Consequently, we need to be cautious about the limitations of clinical-based surveillance programs when interpreting our estimations of the global burden of flavivirus infections (Chandra et al., 2021).

Given the lack of highly effective vaccines for mosquito-borne infections other than yellow fever and Japanese encephalitis, public health interventions have primarily focused on reducing human exposure through vector control (Ferguson, 2018). Over the past century, the use of insecticide-treated nets, long-lasting insecticidal nets, and indoor residual spraying has become the primary and recommended means of mosquito vector control (Wilson et al., 2020). Despite the use of these strategies and the accelerated development of long-lasting insecticidal nets and indoor residual spraying with different compounds, the global burden of mosquito-borne diseases on public health and economies continues to rise (Franklinos et al., 2019). Although the ASIR of flavivirus infections fluctuated worldwide from 2011 to 2021, a significant decrease in the incidence of flavivirus infections was observed in some non-endemic settings during this period, such as Australasia and North Africa and Middle East. Data from the Australia National Notifiable Disease Surveillance System showed that the release of Wolbachia-infected mosquitoes notably decreased local dengue transmission from 2011 to 2019 (Garske et al., 2024). Moreover, there was a substantial decrease in imported dengue notifications amid the COVID-19 pandemic due to travel restrictions. Similarly, several North African countries (Algeria, Libya, Morocco, and Tunisia) have not documented any outbreaks of DENV during 2011–2021 (Petersen et al., 2022). However, the burden of flavivirus infections, as measured by seroprevalence in many countries within the Middle East and North Africa, does not accurately reflect the incidence of flavivirus infections in this area (Humphrey et al., 2016). Despite our results showed an estimated declining trend in the ASIR of flavivirus infections in the Middle East and North Africa, further regional investigations are needed to characterize the epidemiological patterns of flavivirus in this region.

In contrast, we have found that the regions of Asia (except for High-income Asia Pacific) observed a moderate increasing trend in the ASIR of flavivirus infections during the same period. Some studies of these regions have also documented increasing trends in dengue incidence. Results from the National dengue surveillance data for Cambodia revealed that the dengue incidence increased between 2002 and 2020 (Yek et al., 2023). Substantial increases in the number of dengue case have also been found in China for the period 2005–2020 (Yue et al., 2022). The dengue incidence in Southeast Asia is expected to continue rising in the short to medium term; however, this prediction does not consider the effects of COVID-19 restrictions on dengue risk (Colón-González et al., 2023). Chen and colleagues reported a reduced annual dengue incidence across most countries in Latin America and Southeast Asia following the implementation of COVID-19 interventions (Chen et al., 2022). Since it’s unsustainable to continue limiting community mobility in the post-COVID-19 era, vector control interventions remain the best choice for managing flavivirus infections (Sasmono and Santoso, 2022). Notably, greenhouse warming would increase the frequency of disastrous climatic change such as extreme El Niño events, which is highly congenial for the breeding of mosquitoes (Colón-González et al., 2018; Rao et al., 2019). Therefore, sophisticated early warning systems that integrate comprehensive climate indices and provide extended prediction windows enhance global preparedness, enabling more efficient control and prevention of flavivirus epidemics (Chen et al., 2024).

It is encouraging that the global incidence of yellow fever continued to decline between 2011 and 2021, which may be attributed to the inclusion of vaccination against yellow fever in routine infant immunization programs among countries at high risk of yellow fever (Garske et al., 2014). Nevertheless, the global COVID-19 pandemic and other public health priorities have eroded healthcare delivery and access, leading to decreased coverage of yellow fever vaccines (Lindsey et al., 2022). Modeling analysis indicated that achieving and maintaining a 90% population immunity is recommended for the global elimination of yellow fever epidemics (Ndeffo-Mbah and Pandey, 2020). However, vaccination coverage in 2016 was estimated to be substantially below the recommended threshold (Shearer et al., 2018). Meanwhile, the need for a new vaccine against flaviviruses is growing as climate change has increased the number of people exposed to flaviviruses, leading to a limited supply of vaccines produced with existing technology (Lindsey et al., 2022). Children and young adults under 40 years old remain the most affected by flavivirus infections. However, the proportion of cases in older age groups has increased over the years. This changing pattern can be attributed to population aging, as higher proportions of cases in older age groups are observed in higher-income regions, which correspond to lower fertility rates and higher life expectancies in these areas (Schumacher et al., 2024). Our finding is coincident with the impact of demographic transition on the age distribution of dengue in several endemic countries in Southeast Asia (Cummings et al., 2009). Hence, age- and region-appropriate health-care resource planning and allocation should be prioritized.

## 5 Limitations

Our study has some limitations. Firstly, the estimates for flavivirus infections in the GBD Study relies on complex statistical modeling and extrapolation techniques. The definition of cases or measurement approaches may differ geographically and temporally. Therefore, the accuracy and robustness of estimates can fluctuate between regions and health scenarios (Ferrari et al., 2024). Additionally, it’s likely that these burdens are underestimated as mild cases of flavivirus infections frequently pass unnoticed owing to nonspecific symptoms and the limited capacity for surveillance or laboratory diagnostics in numerous vulnerable regions (de Araújo Lobo et al., 2016; Musso et al., 2019; Lindsey et al., 2022). Nonetheless, the GBD 2021 compute an adjustment factor aimed at rectifying underreporting. These adjustment factors were estimated using MR-BRT (meta-regression—Bayesian, regularized, trimmed), which factored in variables such as SDI level and reported incidence rate (Ferrari et al., 2024). Moreover, the flavivirus genus includes many members such as West Nile virus (WNV), Japanese encephalitis virus (JEV), and tick-borne encephalitis virus (TBEV) (Pierson and Diamond, 2020). However, due to limited data, the incidence of these other flaviviruses has not been evaluated in this study. Despite these limitations, our study provides useful information for public health professionals and policymakers to prevent the potential threats posed by the substantial global flavivirus burden.

## 6 Conclusion

In summary, the global burden of flavivirus infections is substantial, with considerable regional and demographic variations in incidence. Our research provides updated evidence of the changing global threat of flavivirus infections and will help decision-makers, healthcare providers, and at-risk communities worldwide to better prepare for and respond to future flavivirus pandemics.

## Data Availability

The original contributions presented in the study are included in the article/Supplementary material, further inquiries can be directed to the corresponding author.

## Footnotes

^1^ http://ghdx.healthdata.org/gbd-results-tool
