# Supplementary material for: The global incidence and trends of three common flavivirus infections (Dengue, yellow fever, and Zika) from 2011 to 2021
Source: Front Microbiol. 2024 Aug 14;15:1458166. doi: 10.3389/fmicb.2024.1458166 (PMC11349664; doi:10.3389/fmicb.2024.1458166)
Supplement: Supplementary file 1 [file Data_Sheet_1.pdf]

Supplementary Materials

The global incidence and trends of three common flavivirus infections (Dengue, Yellow fever, and Zika) from 2011 to 2021

Yuanhao Liang[1,§,*], Xingzhu Dai[2,§]

[1] Clinical Experimental Center, Jiangmen Engineering Technology Research Center of Clinical Biobank and Translational Research, Jiangmen Central Hospital, Jiangmen, China

[2] Department of Stomatology, Guangdong Provincial People's Hospital (Guangdong Academy of Medical Sciences), Southern Medical University, Guangzhou, China

[§] These authors equally contributed to this study.

[*] Corresponding author

This supporting information contains **4** Figures and **2** Tables.

Table of Content

Supplementary Figure S1. The distribution and trend of the number and age-standardized rate of incidence for three prevalent flavivirus (DENV, YFV, and ZIKV) infections, by sex. (A) DENV infection; (B) YFV infection; (C) ZIKV infection. DENV=Dengue virus; YFV=Yellow fever virus; ZIKV=Zika virus.

Supplementary Figure S2. Contribution of DENV, YFV, and ZIKV infections to the absolute number of three prevalent flavivirus (DENV, YFV, and ZIKV) infections globally, in 4 income levels and 21 GBD regions, 2011-2021. DENV=Dengue virus; YFV=Yellow fever virus; ZIKV=Zika virus.

Supplementary Figure S3. The distribution and trend of the number and age-standardized rate of incidence for three prevalent flavivirus (DENV, YFV, and ZIKV) infections across four income levels, by sex. (A) The number and age-standardized rate of incidence from 2011 to 2021; (B) The number and rate of incidence in 2021 across age groups. DENV=Dengue virus; YFV=Yellow fever virus; ZIKV=Zika virus.

Supplementary Figure S4. The number of incident case and age-standardized incidence rate (ASIR) grouped by income level for three prevalent flavivirus (DENV, YFV, and ZIKV) infections, from 2011 to 2021. (A) DENV infection; (B) YFV infection; (C) ZIKV infection. DENV=Dengue virus; YFV=Yellow fever virus; ZIKV=Zika virus.

Supplementary Figure S5. The number of incident case and incidence rate of three prevalent flavivirus (DENV, YFV, and ZIKV) infections across all age groups in 2021, grouped by income level. (A) DENV infection; (B) YFV infection; (C) ZIKV infection. DENV=Dengue virus; YFV=Yellow fever virus; ZIKV=Zika virus.

Supplementary Figure S6. The distribution and trend of the number and age-standardized rate of incidence for three prevalent flavivirus (DENV, YFV, and ZIKV) infections across GBD

regions, by sex. DENV=Dengue virus; YFV=Yellow fever virus; ZIKV=Zika virus.

Supplementary Table S1. The number and age to standardized incidence rates (ASIR, per 100,000) of dengue in 2021, as well as the temporal trends from 2011 to 2019 and the trends from 2019 to 2021.

Supplementary Table S2. The number and age to standardized incidence rates (ASIR, per 100,000) of yellow fever in 2021, as well as the temporal trends from 2011 to 2019 and the trends from 2019 to 2021.

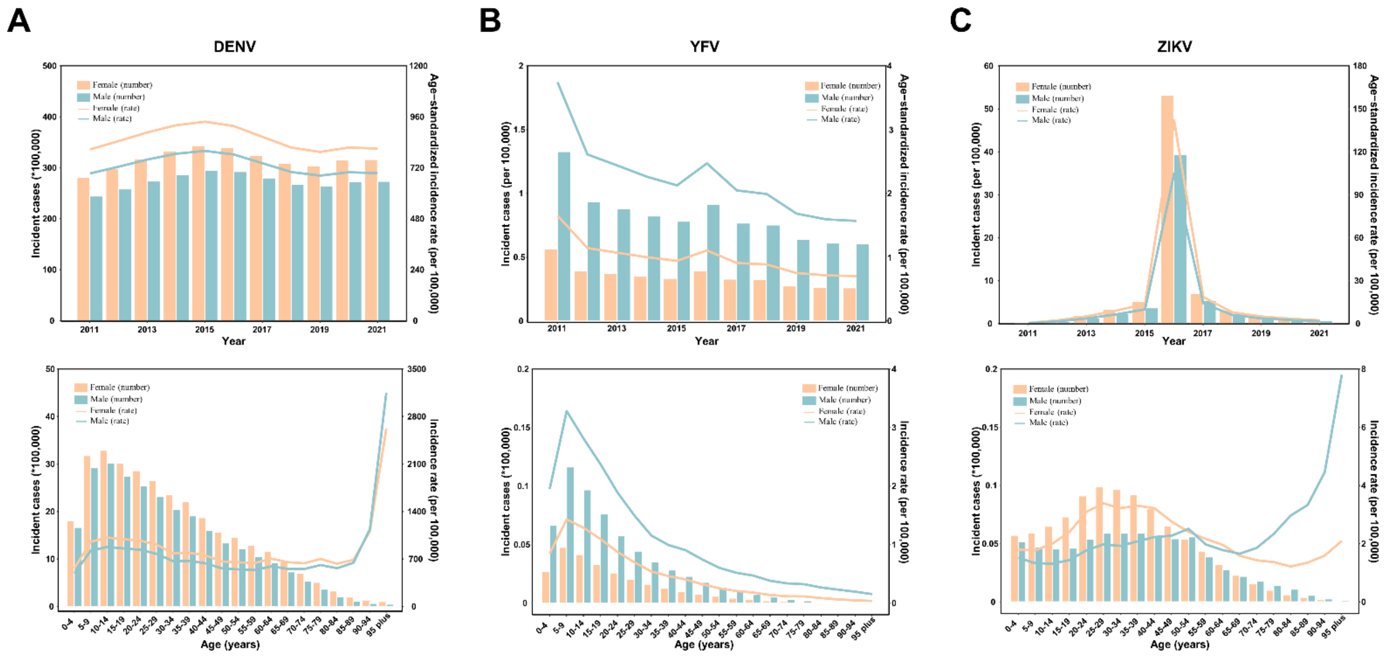

Supplementary Figure S1. The distribution and trend of the number and age-standardized rate of incidence for three prevalent flavivirus (DENV, YFV, and ZIKV) infections, by sex. (A) DENV infection; (B) YFV infection; (C) ZIKV infection. DENV=Dengue virus; YFV=Yellow fever virus; ZIKV=Zika virus.

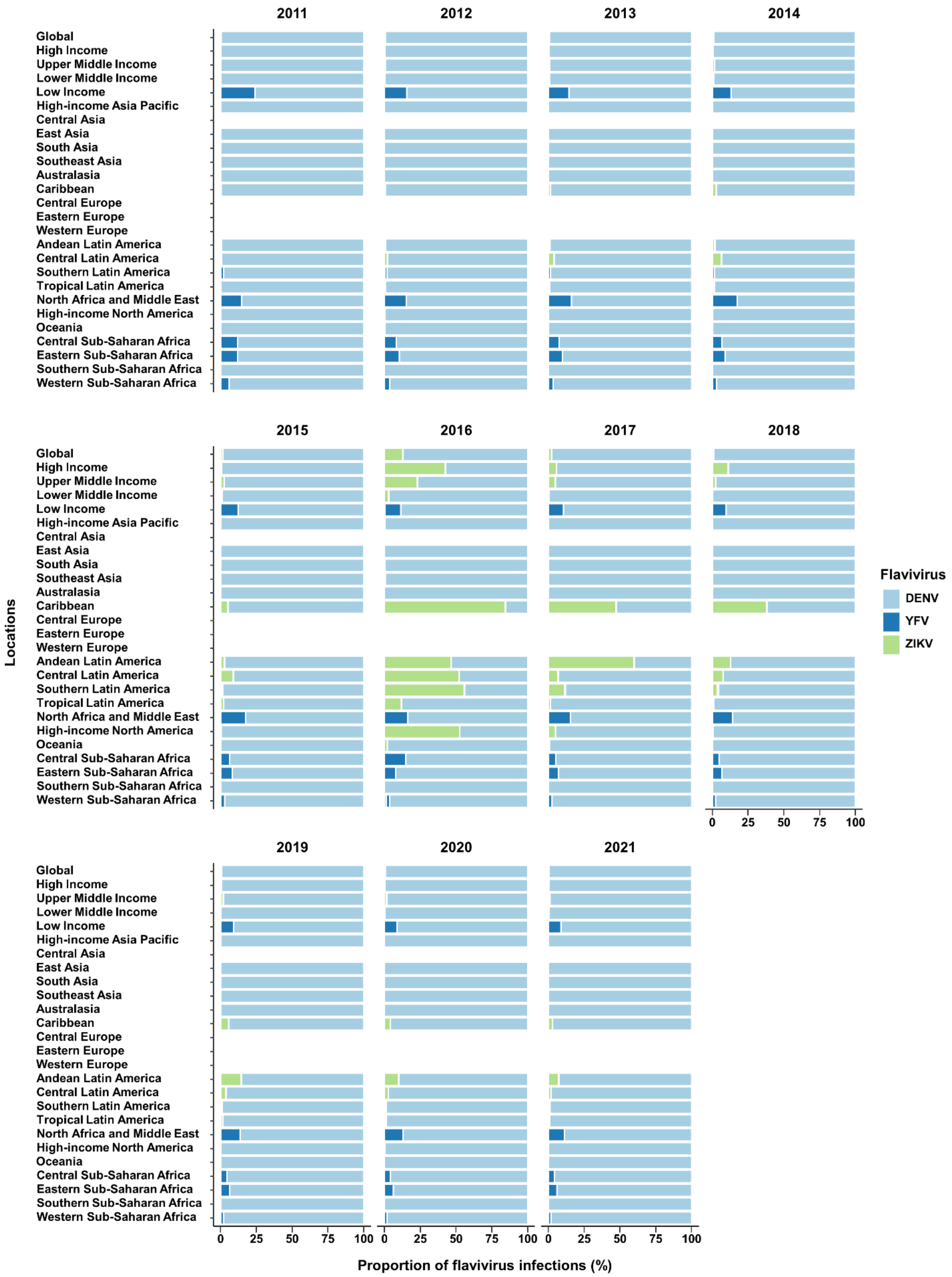

Supplementary Figure S2. Contribution of DENV, YFV, and ZIKV infections to the absolute number of three prevalent flavivirus (DENV, YFV, and ZIKV) infections globally, in 4 income levels and 21 GBD regions, 2011-2021. DENV=Dengue virus; YFV=Yellow fever virus; ZIKV=Zika virus.

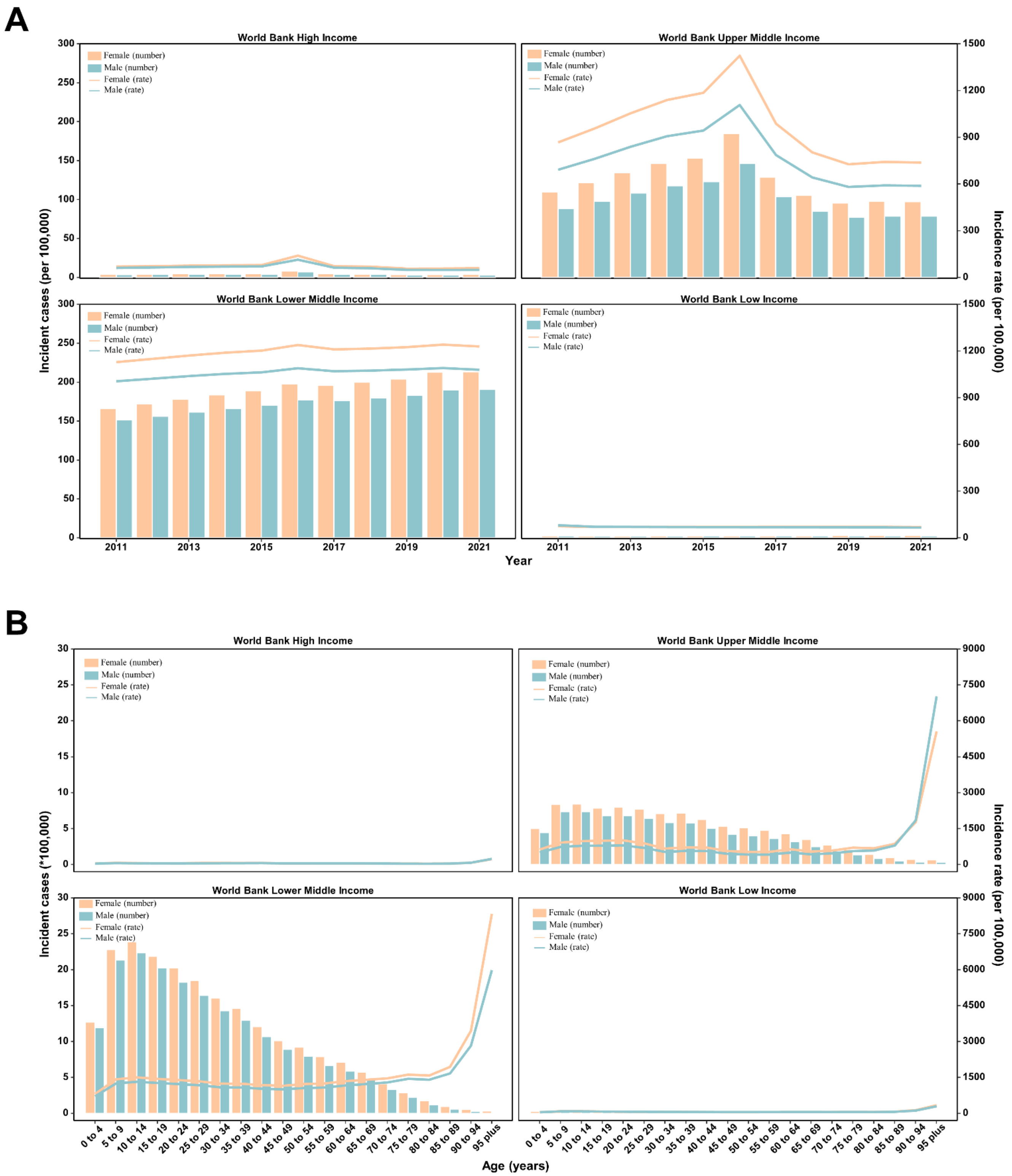

Supplementary Figure S3. The distribution and trend of the number and age-standardized rate of incidence for three prevalent flavivirus (DENV, YFV, and ZIKV) infections across four income levels, by sex. (A) The number and age-standardized rate of incidence from 2011 to 2021; (B) The number and rate of incidence in 2021 across age groups. DENV=Dengue virus; YFV=Yellow fever virus; ZIKV=Zika virus.

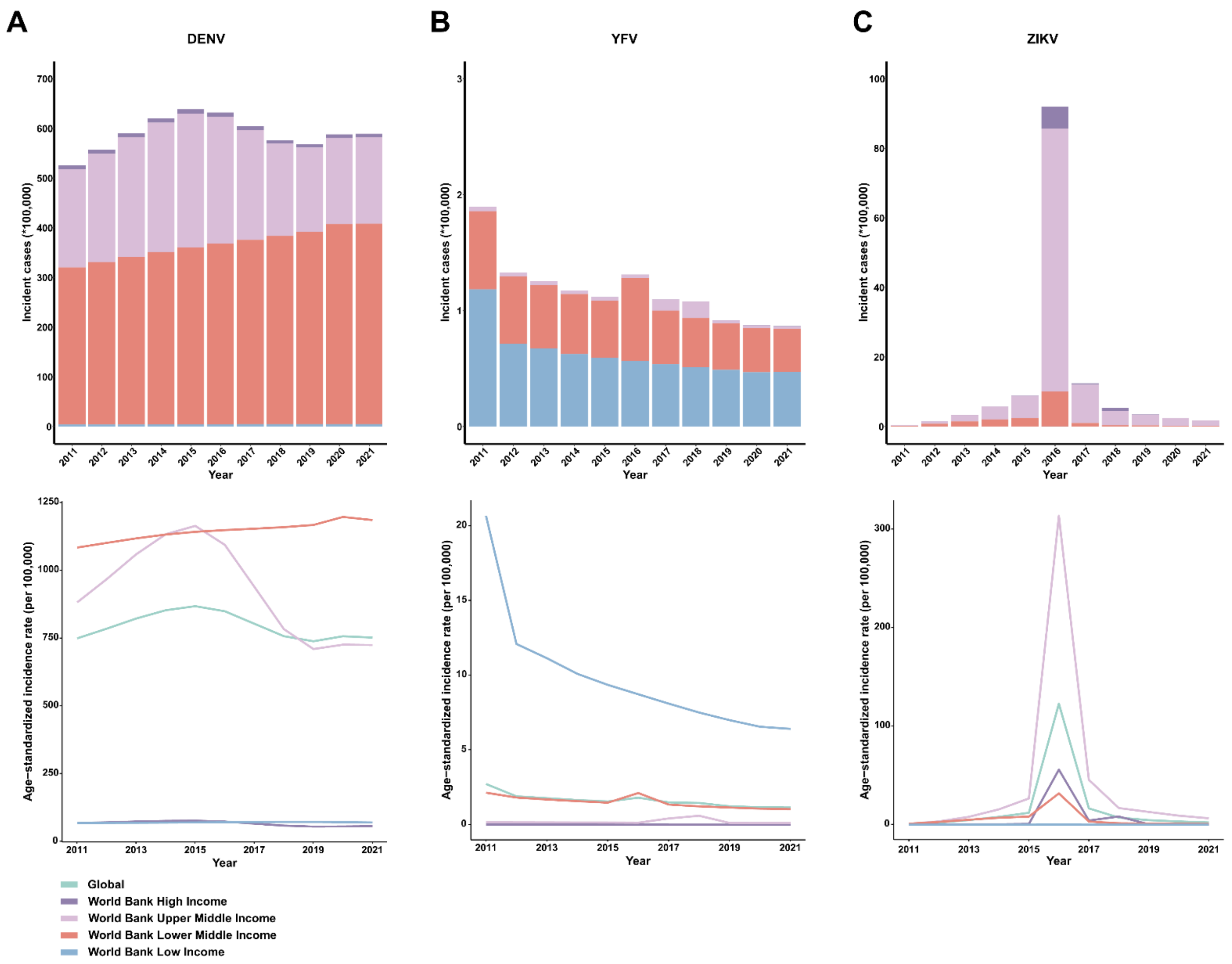

Supplementary Figure S4. The number of incident case and age-standardized incidence rate (ASIR) grouped by income level for three prevalent flavivirus (DENV, YFV, and ZIKV) infections, from 2011 to 2021. (A) DENV infection; (B) YFV infection; (C) ZIKV infection. DENV=Dengue virus; YFV=Yellow fever virus; ZIKV=Zika virus.

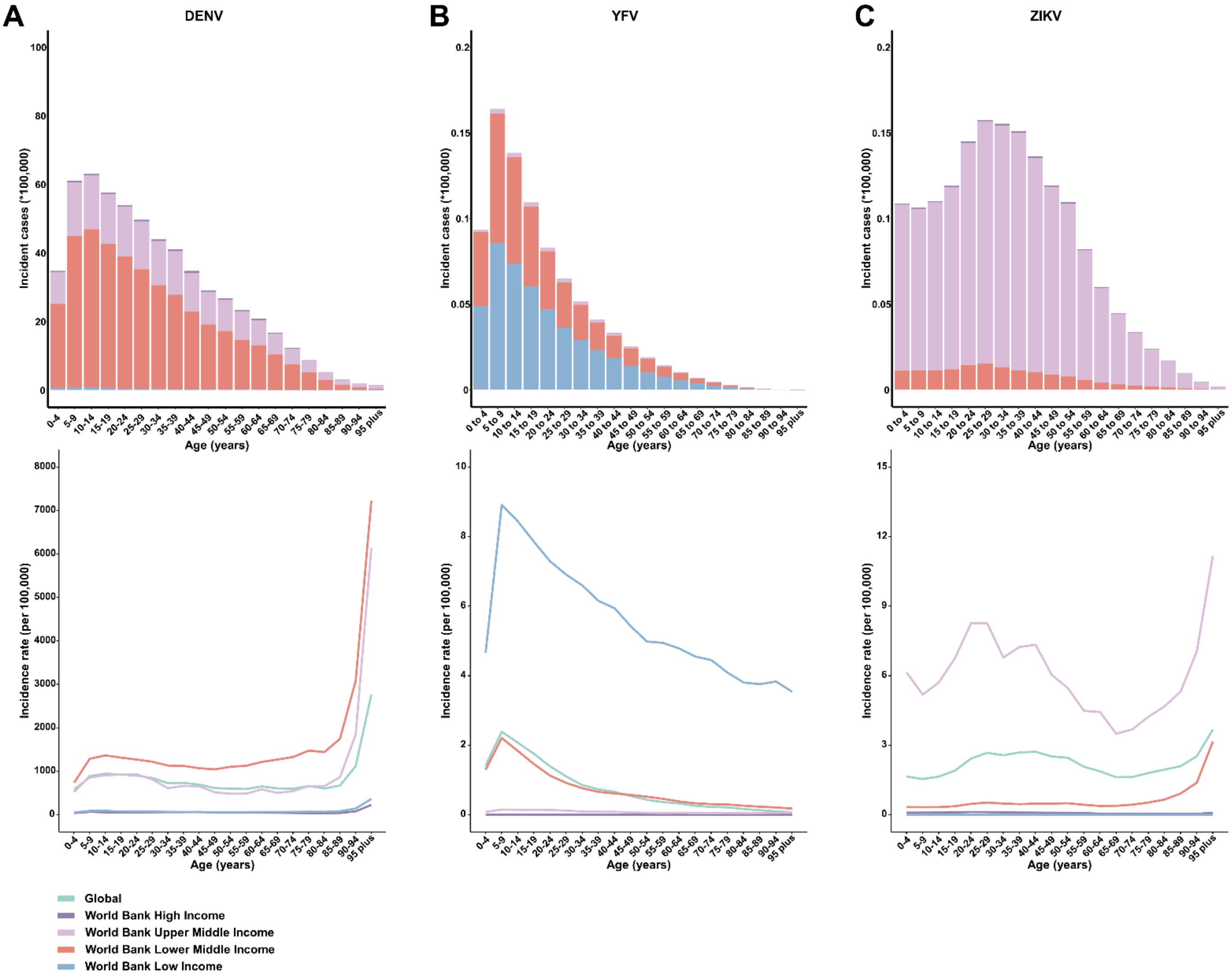

Supplementary Figure S5. The number of incident case and incidence rate of three prevalent flavivirus (DENV, YFV, and ZIKV) infections across all age groups in 2021, grouped by income level. (A) DENV infection; (B) YFV infection; (C) ZIKV infection. DENV=Dengue virus; YFV=Yellow fever virus; ZIKV=Zika virus.

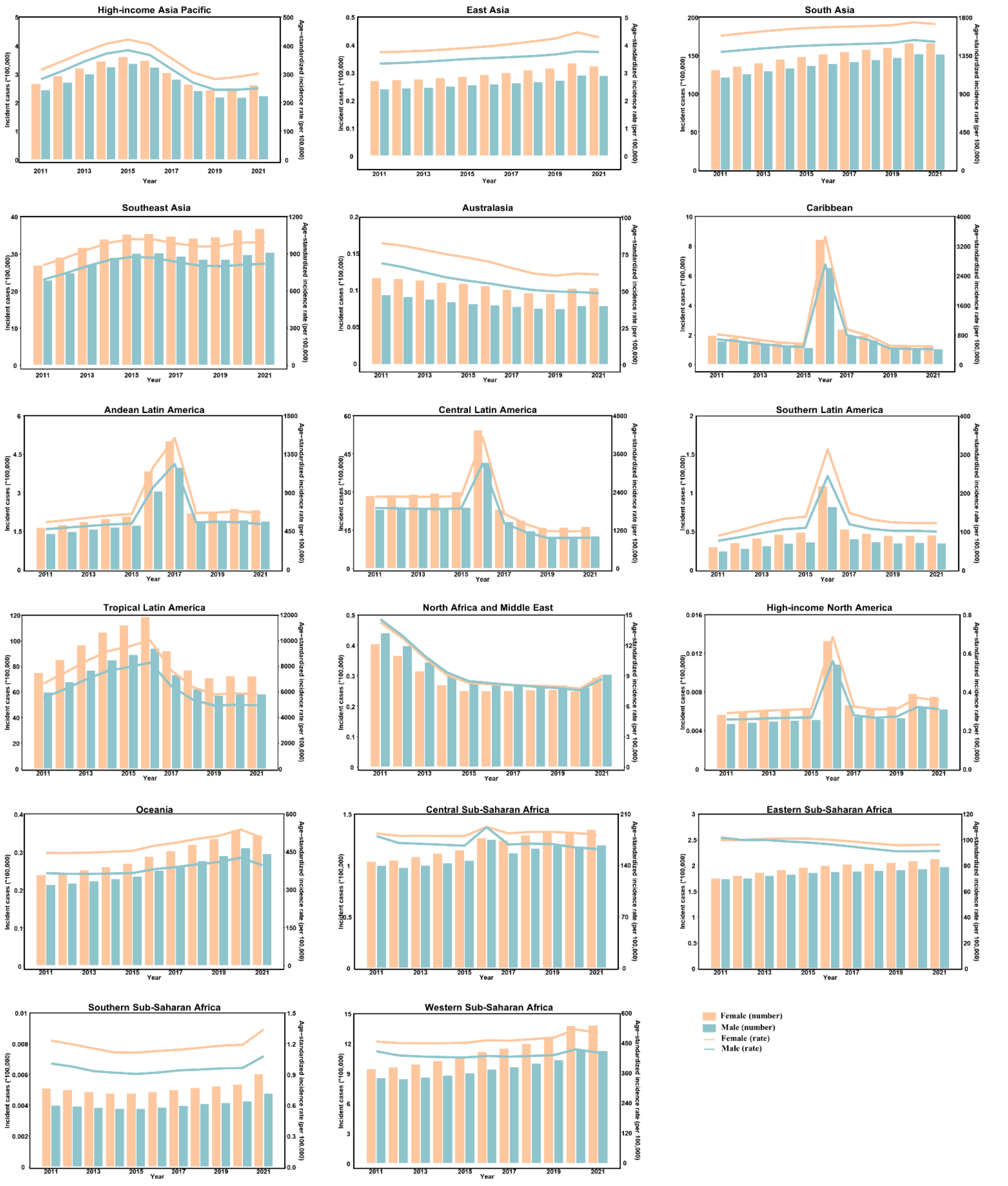

Supplementary Figure S6. The distribution and trend of the number and age-standardized rate of incidence for three prevalent flavivirus (DENV, YFV, and ZIKV) infections across GBD regions, by sex. DENV=Dengue virus; YFV=Yellow fever virus; ZIKV=Zika virus.

Supplementary Table S1. The pooled number and age-standardized incidence rates (ASIR, per 100,000) of three prevalent flavivirus infections in 2021, as well as the temporal trends.

Characteristics	2021		2011−2016		2016−2019		2019−2021	
	Case number	ASIR per 100,000	Percent change (%)	EAPC (95% CI)	Percent change (%)	EAPC (95% CI)	Percent change (%)	EAPC (95% CI)
**Overall**	59,220,428	715.69	37.5	5.08 (4.12 to 6.05)	−8.8	−8.37 (−12.46 to −4.08)	3.5	0.69 (−0.96 to 2.37)
**Sex**								
Male	27,480,266	661.04	20.6	4.81 (3.96 to 5.67)	−20	−7.93 (−11.75 to −3.95)	3.2	0.58 (−0.96 to 2.14)
Female	31,740,162	771.58	23.1	5.31 (4.25 to 6.39)	−22.2	−8.77 (−13.11 to −4.2)	3.7	0.79 (−0.96 to 2.57)
**World Bank classification**								
High-income	686,716	54.5	96.8	10.65 (2.61 to 19.32)	−25.2	−23.22 (−34.87 to −9.5)	3.8	0.86 (−0.02 to 1.76)
Upper-middle-income	17,601,004	661.42	67.2	9.51 (7.91 to 11.14)	−34.5	−19.66 (−25.86 to −12.94)	1.9	0.72 (−0.73 to 2.18)
Lower-middle-income	40,368,521	1154.6	18	1.67 (1.48 to 1.86)	10.8	−0.26 (−1.17 to 0.66)	4.2	0.06 (−1.12 to 1.25)
Low-income	543,556	68.15	1.3	−1.79 (−3.28 to −0.28)	11.9	−0.27 (−0.43 to −0.11)	1.1	−1.09 (−2.29 to 0.12)
**GBD regions**								

High-income Asia Pacific	485,712	278.97	31.1	5.76 (3.14 to 8.45)	−30.8	−12.15 (−13.89 to −10.38)	4.3	2.49 (1.41 to 3.58)
Central Asia	0	0	NA	NA	NA	NA	NA	NA
East Asia	61,440	3.99	7.8	1.19 (1.06 to 1.32)	10.6	1.79 (1.6 to 1.97)	4.1	0.96 (−2.82 to 4.88)
South Asia	31,812,189	1624.14	15.2	1.23 (1.03 to 1.42)	10.7	0.5 (0.44 to 0.55)	3.2	0.41 (−1.47 to 2.33)
Southeast Asia	6,728,444	914.64	32	4.95 (3.37 to 6.55)	0.1	−2.29 (−3.34 to −1.22)	6.5	1.39 (0.43 to 2.35)
Australasia	18,448	55.42	−11.5	−3.88 (−4.09 to −3.67)	−12.3	−4.15 (−5.16 to −3.13)	6.7	−0.2 (−1.45 to 1.07)
Caribbean	233,544	458.13	319.4	18.98 (−12.61 to 61.98)	−10	−44.15 (−59.25 to −23.45)	−2.3	−1.48 (−3.19 to 0.26)
Central Europe	0	0	NA	NA	NA	NA	NA	NA
Eastern Europe	0	0	NA	NA	NA	NA	NA	NA
Western Europe	0	0	NA	NA	NA	NA	NA	NA
Andean Latin America	422,524	601.73	126.8	12.24 (2.69 to 22.68)	14.2	−22.19 (−40.85 to 2.34)	1.1	−1.43 (−3.76 to 0.94)
Central Latin America	2,937,860	1090.39	85.5	8.62 (−1.12 to 19.31)	−45.8	−32.55 (−46.27 to −15.33)	2.1	0.38 (−0.55 to 1.31)
Southern Latin America	80,946	112.9	246.2	21.95 (9.47 to 35.85)	−1.2	−24.51 (−39.64 to −5.6)	0	−0.75 (−0.84 to −0.66)
Tropical Latin America	13,125,808	5464.54	57.6	8.47 (6.61 to 10.35)	−33	−16.26 (−20.53 to −11.76)	2	0.23 (−0.97 to 1.44)
North Africa and Middle East	60,050	9.01	−37.9	−11.45 (−13.59 to −9.25)	−8.2	−1.43 (−1.51 to −1.34)	14.5	5.88 (−4.14 to 16.95)
High-income North America	1,378	0.33	132.2	12.92 (0.18 to 27.3)	5.2	−20.7 (−37.5 to 0.62)	16.3	6.75 (−5.4 to 20.46)

Oceania	63,970	452.49	18.9	0.89 (0.17 to 1.61)	27.5	2.6 (2.4 to 2.8)	2.3	−1.26 (−7.4 to 5.3)
Central Sub-Saharan Africa	255,340	174.17	23.7	0.69 (−1.26 to 2.68)	17.9	−2.28 (−5.19 to 0.73)	0.9	−1.46 (−1.71 to −1.21)
Eastern Sub-Saharan Africa	412,043	94.18	10.7	−0.41 (−0.61 to −0.21)	5.9	−1.63 (−1.67 to −1.59)	3.4	0.16 (0.11 to 0.21)
Southern Sub-Saharan Africa	1,087	1.24	−4	−1.91 (−2.72 to −1.1)	9.7	1.56 (1.32 to 1.81)	15.3	6.12 (−0.01 to 12.62)
Western Sub-Saharan Africa	2,519,644	489.28	14.3	−0.27 (−0.89 to 0.35)	19.7	0.57 (0.08 to 1.07)	9.7	1.58 (−3.07 to 6.45)

Supplementary Table S2. Global incidence rates (per 100,000 population) of three common flavivirus infections (Dengue, Yellow fever, and Zika) in 2021, by age group.

Locations	Dengue	Yellow fever	Zika
**Global**			
0-4	527.54 (131.16 to 978.12)	1.42 (0.56 to 3.36)	1.65 (0.61 to 4.22)
5-9	888.19 (220 to 1636.7)	2.39 (0.87 to 5.45)	1.55 (0.54 to 4.08)
10-14	945.96 (229.24 to 1751.97)	2.08 (0.81 to 4.38)	1.65 (0.58 to 4.44)
15-19	923.17 (223.25 to 1713.26)	1.75 (0.7 to 3.89)	1.91 (0.7 to 4.79)
20-24	904.03 (224.69 to 1661.77)	1.39 (0.54 to 3.22)	2.43 (0.89 to 5.95)
25-29	844.88 (217.84 to 1537.51)	1.1 (0.42 to 2.41)	2.68 (0.96 to 6.87)
30-34	727.69 (191.2 to 1316.48)	0.85 (0.32 to 1.9)	2.57 (0.89 to 6.66)
35-39	733.7 (197.58 to 1323.32)	0.73 (0.28 to 1.61)	2.69 (0.93 to 6.83)
40-44	694.24 (193.63 to 1245.46)	0.66 (0.25 to 1.53)	2.73 (0.94 to 7.08)
45-49	613.41 (173.72 to 1097.87)	0.54 (0.21 to 1.17)	2.52 (0.93 to 6.66)
50-54	600.19 (175.24 to 1068.86)	0.43 (0.17 to 0.94)	2.46 (0.92 to 6.56)
55-59	591.24 (176.64 to 1050.51)	0.36 (0.14 to 0.81)	2.08 (0.75 to 5.16)
60-64	651.65 (193.46 to 1154.36)	0.32 (0.12 to 0.77)	1.88 (0.67 to 4.86)
65-69	606.87 (178.49 to 1078.45)	0.26 (0.1 to 0.59)	1.63 (0.58 to 4.3)
70-74	598.85 (173.53 to 1070.44)	0.22 (0.08 to 0.53)	1.63 (0.56 to 4.28)
75-79	661.43 (192.89 to 1179.99)	0.21 (0.08 to 0.49)	1.81 (0.63 to 5.13)
80-84	604.01 (178.69 to 1078.39)	0.16 (0.06 to 0.34)	1.95 (0.67 to 4.96)

85-89	672.86 (209.13 to 1195.87)	0.13 (0.05 to 0.31)	2.1 (0.69 to 5.57)
90-94	1120.3 (370.02 to 1998.35)	0.1 (0.04 to 0.23)	2.53 (0.82 to 6.89)
95 plus	2768.78 (1012.68 to 4885.7)	0.07 (0.03 to 0.16)	3.69 (1.03 to 9.68)
**World Bank High Income**			
0-4	42.15 (13.56 to 86.43)	0 (0 to 0.01)	0.1 (0.01 to 0.38)
5-9	69.46 (22.02 to 142.2)	0.01 (0 to 0.02)	0.1 (0.01 to 0.37)
10-14	59 (18.97 to 117.29)	0 (0 to 0.01)	0.1 (0.01 to 0.37)
15-19	53.61 (16.64 to 106.96)	0 (0 to 0.01)	0.11 (0.02 to 0.4)
20-24	52.36 (16.13 to 105.72)	0 (0 to 0.01)	0.12 (0.02 to 0.45)
25-29	61.05 (18.09 to 127.72)	0 (0 to 0.01)	0.12 (0.02 to 0.42)
30-34	64.13 (18.42 to 137.57)	0 (0 to 0.01)	0.11 (0.02 to 0.38)
35-39	64.45 (18.31 to 139.39)	0 (0 to 0.01)	0.1 (0.01 to 0.35)
40-44	67.94 (18.33 to 147.38)	0 (0 to 0.01)	0.09 (0.01 to 0.33)
45-49	50.64 (14 to 107.98)	0 (0 to 0.01)	0.09 (0.01 to 0.32)
50-54	50.18 (13.63 to 107.23)	0 (0 to 0.01)	0.09 (0.01 to 0.33)
55-59	51.5 (13.6 to 110.67)	0	0.07 (0.01 to 0.24)
60-64	51.94 (13.54 to 112)	0	0.06 (0.01 to 0.19)
65-69	50.12 (12.9 to 107.77)	0	0.05 (0.01 to 0.18)
70-74	45.1 (11.54 to 95.86)	0	0.05 (0.01 to 0.16)
75-79	41.11 (10.66 to 85.9)	0	0.05 (0.01 to 0.16)
80-84	39.62 (10.18 to 82.84)	0	0.05 (0.01 to 0.16)
85-89	43.76 (11.11 to 91.83)	0	0.05 (0.01 to 0.16)
90-94	79.72 (20.14 to 167.46)	0	0.05 (0.01 to 0.18)

95 plus	227.21 (50.27 to 501.33)	0	0.08 (0.01 to 0.29)
**World Bank Upper Middle Income**			
0-4	592.49 (263.09 to 1051.79)	0.08 (0.03 to 0.19)	6.14 (2.23 to 16.65)
5-9	860.49 (389.5 to 1519.29)	0.15 (0.05 to 0.43)	5.19 (1.76 to 14.05)
10-14	914.45 (417.44 to 1608.78)	0.15 (0.05 to 0.37)	5.72 (1.91 to 16.34)
15-19	923.32 (421.2 to 1625.03)	0.15 (0.05 to 0.37)	6.76 (2.46 to 17.65)
20-24	934.88 (421.76 to 1658.67)	0.14 (0.05 to 0.34)	8.27 (2.98 to 21.16)
25-29	816.38 (370.23 to 1449.31)	0.12 (0.04 to 0.33)	8.26 (2.78 to 22.4)
30-34	616.12 (277.07 to 1098.83)	0.09 (0.03 to 0.26)	6.79 (2.28 to 18.1)
35-39	667.18 (292.2 to 1198)	0.09 (0.03 to 0.25)	7.24 (2.43 to 19.11)
40-44	658.32 (283.11 to 1188.15)	0.09 (0.03 to 0.23)	7.34 (2.49 to 19.11)
45-49	521.05 (226.71 to 938.87)	0.07 (0.02 to 0.16)	6.04 (2.18 to 16.41)
50-54	488.54 (212.64 to 880.32)	0.06 (0.02 to 0.14)	5.47 (1.95 to 14.71)
55-59	490.01 (211.12 to 886.61)	0.05 (0.02 to 0.13)	4.48 (1.57 to 11.38)
60-64	590.27 (251.62 to 1068.7)	0.06 (0.02 to 0.16)	4.43 (1.56 to 11.87)
65-69	505.67 (214.13 to 918.96)	0.05 (0.01 to 0.11)	3.5 (1.2 to 9.15)
70-74	548.15 (231.09 to 993.6)	0.05 (0.01 to 0.12)	3.68 (1.23 to 9.96)
75-79	662.6 (281.74 to 1200)	0.05 (0.02 to 0.14)	4.23 (1.41 to 11.72)
80-84	664.15 (278.39 to 1205.87)	0.05 (0.01 to 0.13)	4.68 (1.52 to 11.8)
85-89	870.93 (366.68 to 1589.85)	0.05 (0.02 to 0.15)	5.32 (1.7 to 13.92)
90-94	1850.04 (760.79 to 3371.05)	0.06 (0.02 to 0.15)	7.05 (2.2 to 19.3)
95 plus	6146.21 (2597.15 to 11254.39)	0.07 (0.02 to 0.17)	11.19 (2.95 to 30.9)
**World Bank Lower Middle Income**			

0-4	736.13 (114.19 to 1466.27)	1.3 (0.49 to 2.81)	0.34 (0.07 to 1.05)
5-9	1288.53 (201.72 to 2537.2)	2.21 (0.87 to 4.92)	0.33 (0.08 to 1.03)
10-14	1367.28 (206.74 to 2714.87)	1.83 (0.71 to 3.81)	0.34 (0.08 to 1.05)
15-19	1315.22 (194.97 to 2619.82)	1.46 (0.59 to 3.26)	0.37 (0.09 to 1.23)
20-24	1271.14 (191.98 to 2524.77)	1.12 (0.44 to 2.41)	0.48 (0.11 to 1.54)
25-29	1221.97 (193.66 to 2403.98)	0.91 (0.35 to 1.97)	0.53 (0.12 to 1.68)
30-34	1132.63 (183.48 to 2203.76)	0.76 (0.31 to 1.65)	0.49 (0.11 to 1.48)
35-39	1124.85 (185.73 to 2178.28)	0.66 (0.25 to 1.48)	0.46 (0.11 to 1.47)
40-44	1071.12 (186.24 to 2058.87)	0.61 (0.24 to 1.33)	0.49 (0.11 to 1.49)
45-49	1046.28 (183.07 to 2004.46)	0.57 (0.23 to 1.24)	0.49 (0.11 to 1.55)
50-54	1104.83 (201.03 to 2105.61)	0.52 (0.2 to 1.1)	0.5 (0.11 to 1.63)
55-59	1128.69 (209.66 to 2140.09)	0.46 (0.17 to 0.96)	0.44 (0.09 to 1.36)
60-64	1216.72 (224.76 to 2305.55)	0.38 (0.14 to 0.87)	0.39 (0.08 to 1.22)
65-69	1271.76 (229.69 to 2419.9)	0.33 (0.13 to 0.7)	0.39 (0.07 to 1.33)
70-74	1330.16 (226.76 to 2554.28)	0.3 (0.13 to 0.67)	0.44 (0.08 to 1.63)
75-79	1477.34 (245.75 to 2843.49)	0.29 (0.11 to 0.64)	0.53 (0.1 to 1.95)
80-84	1442.91 (238.83 to 2771.34)	0.26 (0.1 to 0.56)	0.66 (0.12 to 2.39)
85-89	1751.38 (306.13 to 3324.19)	0.23 (0.09 to 0.51)	0.92 (0.17 to 3.5)
90-94	3085.73 (547.66 to 5842.97)	0.21 (0.08 to 0.46)	1.39 (0.22 to 5.52)
95 plus	7233.15 (1347.85 to 13622.44)	0.17 (0.07 to 0.38)	3.16 (0.34 to 14.62)
**World Bank Low Income**			
0-4	52.85 (2.66 to 204.88)	4.66 (1.61 to 11.81)	0
5-9	88.92 (4.42 to 347.36)	8.92 (2.94 to 21.07)	0

10-14	88.5 (4.44 to 347.21)	8.44 (2.98 to 19.13)	0
15-19	79.41 (3.99 to 310.4)	7.85 (2.77 to 18.94)	0
20-24	74.49 (3.68 to 291.19)	7.29 (2.61 to 17.56)	0
25-29	71.9 (3.53 to 285.32)	6.91 (2.44 to 17.11)	0
30-34	67.07 (3.33 to 271.01)	6.6 (2.33 to 15.5)	0
35-39	65.45 (3.31 to 262.37)	6.15 (2.07 to 14.07)	0
40-44	61.01 (3.06 to 239.75)	5.94 (2.03 to 14.67)	0
45-49	57.38 (2.84 to 226.9)	5.42 (1.92 to 12.41)	0
50-54	58.22 (2.86 to 237.83)	4.98 (1.86 to 10.84)	0
55-59	61.33 (3.03 to 253.96)	4.94 (1.76 to 11.5)	0
60-64	64.82 (3.26 to 270.35)	4.78 (1.69 to 11.48)	0
65-69	65.66 (3.38 to 272.29)	4.55 (1.59 to 11.4)	0
70-74	66.44 (3.56 to 277.48)	4.45 (1.43 to 11.53)	0
75-79	69.54 (3.83 to 282.38)	4.09 (1.44 to 10.25)	0
80-84	67.48 (3.85 to 268.53)	3.8 (1.38 to 8.15)	0
85-89	80.03 (4.63 to 317.1)	3.76 (1.33 to 9.15)	0
90-94	144.1 (8.22 to 572.81)	3.83 (1.33 to 10.24)	0
95 plus	365.13 (20.55 to 1422.19)	3.54 (1.09 to 9.08)	0
